# Endovascular Therapy Is Effective for Leriche Syndrome with Deep Vein Thrombosis

**DOI:** 10.1155/2015/395205

**Published:** 2015-05-03

**Authors:** Tasuku Higashihara, Nobuo Shiode, Tomoharu Kawase, Hiromichi Tamekiyo, Masaya Otsuka, Tomokazu Okimoto, Yasuhiko Hayashi

**Affiliations:** Cardiovascular Center, Division of Cardiology, Akane Foundation, Tsuchiya General Hospital, 3-30 Nakajima-cho, Naka-ku, Hiroshima 730-8655, Japan

## Abstract

A 65-year-old man presented to our hospital due to intermittent claudication and swelling in his left leg. He had Leriche syndrome and deep vein thrombosis. We performed endovascular therapy (EVT) for Leriche syndrome, and a temporary filter was inserted in the inferior vena cava. He received anticoagulation therapy for deep vein thrombosis. The stenotic lesion in the terminal aorta was stented with an excellent postprocedural angiographic result and dramatic clinical improvement after EVT. This case suggests that EVT can be a treatment for Leriche syndrome.

## 1. Introduction

Leriche syndrome is a chronic obstruction of the aortic bifurcation, extending to both the infrarenal aorta and the common iliac arteries, and is classically associated with a triad of symptoms comprising intermittent claudication, absent or diminished peripheral pulses, and erectile dysfunction in men [1]. The disease manifested as Leriche syndrome is nowadays categorized as a type D aortoiliac lesion by the Trans-Atlantic Inter-Society Consensus for the Management of Peripheral Arterial Disease (TASC II) [2]. May-Thurner syndrome is an iliac vein compression syndrome in which anatomic compression of the left common iliac vein by the overlying right common iliac artery occurs, therefore, resulting in development of left lower extremity deep vein thrombosis (DVT) [3].

Here we report a case of Leriche syndrome with DVT resembling May-Thurner syndrome that we treated with endovascular therapy (EVT).

## 2. Case Report

A 65-year-old male presented to our hospital due to intermittent claudication of both legs and swelling in his left leg. The claudication had started 4 weeks earlier and was ongoing at the time of presentation, and the pain had worsened. His left lower limb had become swollen 2 weeks before admission. On admission, the pulsation of his bilateral femoral, popliteal, and anterior tibial arteries was weak. The ankle-brachial index (ABI) was significantly low bilaterally.

**Figure 1:**
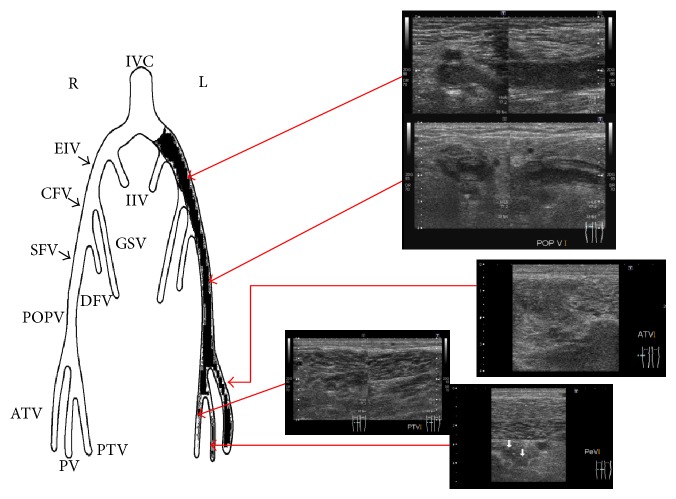
Ultrasonography showed the left iliac vein was occluded with a thrombus.

**Figure 2:**
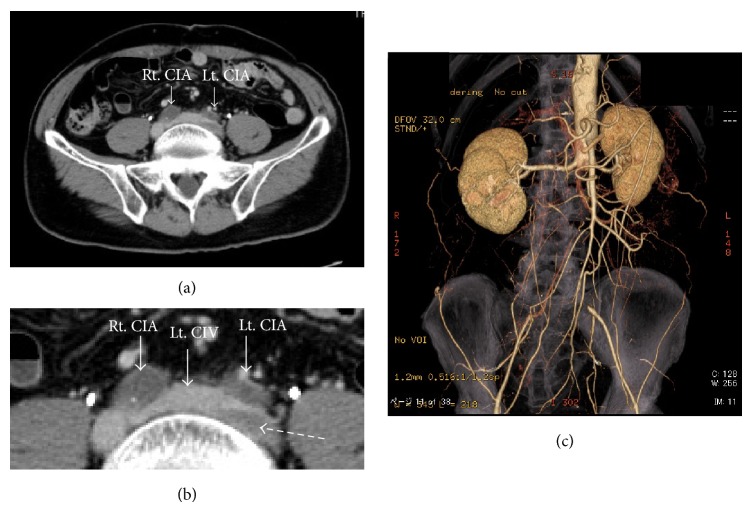
(a) The left common iliac vein (Lt. CIV) was compressed by the left common iliac artery (Lt. CIA) resulting in formation of a venous thrombus at this point. (b) The thrombus was observed in left common iliac vein (dotted arrow). (c) CT showed that the terminal aorta was occluded from the level of renal artery to the bilateral common iliac arteries.

Ultrasonography showed that there were massive thrombi in the veins extending from the left external iliac vein to the left popliteal vein (Figure 1). At the same time, there was no discernible arterial blood flow from the infrarenal abdominal aorta to both the common iliac arteries. Further, the inside echo of the occlusion site was low, so the occlusion seemed to be due to a thrombus. Pulmonary thromboembolism was ruled out by echocardiographic examination. The echocardiography showed normal left ventricular function (left ventricular ejection fraction 73%), mild left ventricular hypertrophy, and no tricuspid regurgitation. We considered that if pulmonary thromboembolism had occurred, the grade was not severe. In the computed tomography (CT) findings, the terminal aorta was occluded with thrombus and there was a venous thrombus in his left iliac vein that appeared in the proximal site that was compressed by the left common iliac artery (Figure 2). The patient was diagnosed with Leriche syndrome accompanied with DVT. His thyroid function tests were normal and his hypercoagulable workup including serum protein C, protein S, anti-cardiolipin antibodies, and lupus anticoagulant antibody was found to be negative.

On day 1, a temporary inferior vena cava (IVC) filter was inserted to prevent pulmonary embolism. Oral warfarin administration and intravenous heparin infusion were started for DVT. Oral cilostazol (200 mg/day) was started for ischemia of the lower extremities. Coronary angiography (CAG) and aortography were done to plan the treatment strategy. There was 75% stenosis in the middle of the left circumflex coronary artery; however, he had no chest symptom, so we decided to continue observation with oral medication.

**Figure 3:**
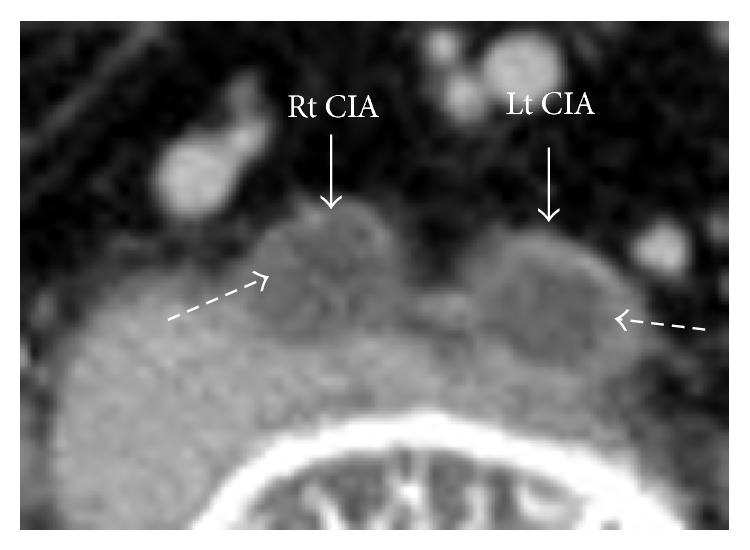
CT showed that the bilateral common iliac arteries were occluded with thrombus. The high density area was observed in low density area (dotted arrows).

We planned the treatment strategy as follows. CT showed that the terminal aorta was occluded with thrombus. The high density area was observed in low density area (Figure 3). It seemed that thrombus was comparatively fresh. The patient had received abdominal surgery for intestinal obstruction about 20 years before, so the adhesion of abdominal organs was suspected. Therefore, we decided to perform EVT for Leriche syndrome.

**Figure 4:**
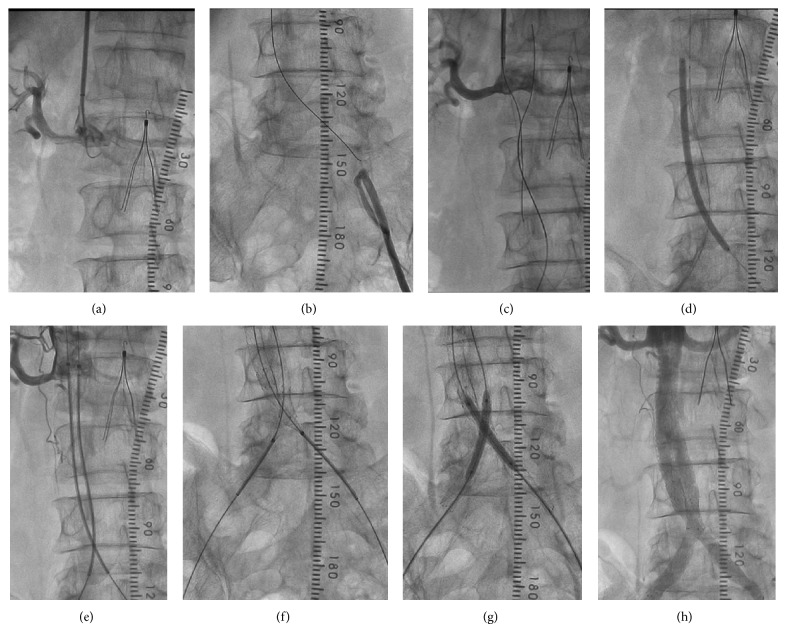
(a) The proximal fibrous cap of the occlusion site of terminal aorta was penetrated by using a multipurpose catheter and a 0.035-inch Radifocus guidewire. (b) Treasure XS12 was crossed from the aorta to the left external iliac artery. (c) Another Treasure XS12 was advanced from the right femoral artery to the aorta. (d) The occluded segments of the bilateral iliac arteries were predilated with either of the two 4.0 mm balloons. (e, f) An Epic 10 mm (98 mm) and a 10 mm (80 mm) stent were inserted from the right common iliac artery and advanced to the aorta. Also, an Epic 10 mm (98 mm) and a 10 mm (60 mm) stent were inserted in the left iliac artery from the left common iliac artery and advanced to the aorta. (g) The postdilatation of the bilateral stents was performed simultaneously with two 5.0 mm balloons. (h) The final angiogram.

**Figure 5:**
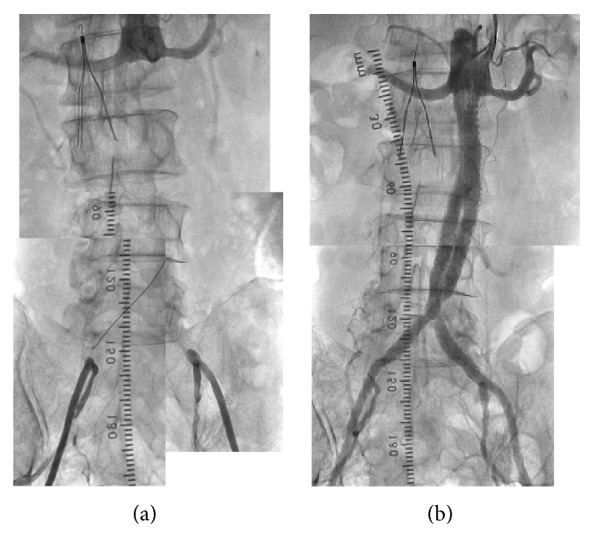
(a) Aortography showed that the terminal aorta was occluded from the level of the renal artery to the bilateral common iliac arteries. (b) After EVT, aortography showed that the bilateral common iliac arterial flow was restored. EVT: endovascular therapy.

Following local anesthesia, a 90 cm 6F sheath was inserted from the right brachial artery and advanced to the distal abdominal aorta. The 6F sheath was placed in the right femoral artery and a 7F sheath was placed in the left FA. The proximal fibrous cap of the occlusion site of terminal aorta was penetrated by using a multipurpose catheter and a 0.035-inch Radifocus guidewire (GW) (Terumo Corp., Japan). Then the Radifocus GW was exchanged for a Treasure XS12 (Asahi Intec Co., Aichi, Japan) and crossed from the aorta to the left external iliac artery. A Corsair PV (Asahi Intec Co., Aichi, Japan) was crossed from the left femoral artery to the aorta by the Rendez-Vous Technique, and we exchanged the GW for a Runthrough Ph guidewire (Terumo Corp., Japan). Next, we crossed the Corsair PV and Treasure XS12 from the right femoral artery to the aorta and exchanged the GW for the Runthrough Ph. The intraluminal position of the GW was confirmed by the intravascular ultrasound. After the two wires were successfully passed through, the thrombi were aspirated using a Thrombuster II for 8F aspiration catheter (Kaneka Medix Corp., Japan) and next with a 6F guide catheter Heartrail BL3.5 (Terumo Corp., Japan). The occluded segments of the bilateral iliac arteries were predilated simultaneously with either of the two 4.0 mm balloons. After that, an Epic 10 mm (98 mm) and a 10 mm (80 mm) stent (Boston Scientific, Natick, MA, USA) were inserted from the right common iliac artery and advanced to the aorta. Also, an Epic 10 mm (98 mm) and a 10 mm (60 mm) stent were inserted in the left iliac artery from the left common iliac artery and advanced to the aorta, and postdilatation of the bilateral stents was performed simultaneously with two 5.0 mm balloons. The final angiogram showed no thromboembolism in the distal arteries (Figures 4 and 5).

**Figure 6:**
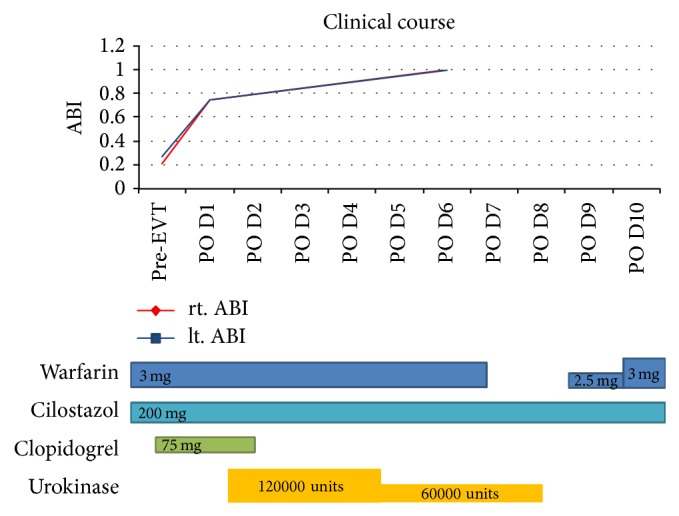
Clinical course after endovascular therapy. ABI: ankle-brachial index, EVT: endovascular therapy, and POD: postoperative day.

**Figure 7:**
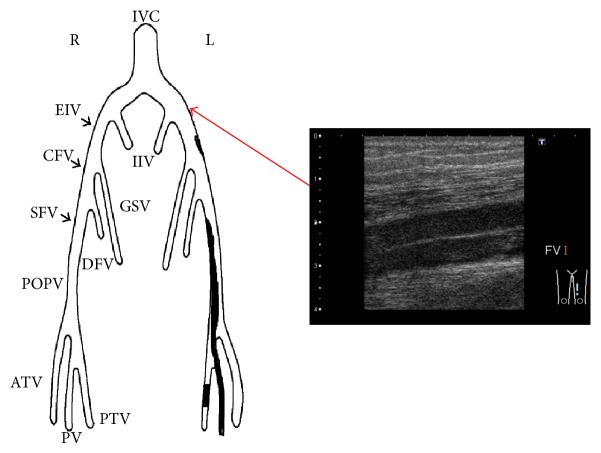
After anticoagulant therapy and EVT, the venous thrombi in the left leg decreased as seen in the ultrasonography of leg veins.

The clinical course after the EVT is showed in Figure 6. The ABI dramatically improved (right 0.21 to 0.97 and left 0.27 to 0.98). On day 12 after EVT, the ultrasonography revealed that the venous thrombi in his left leg had decreased (Figure 7). On day 17 after EVT, the IVC filter was removed and the patient was discharged on the 28th hospital day (20th day after EVT).

## 3. Discussion

In general, surgical treatment has been recommended as a revascularization therapy for Leriche syndrome [2]. Recently the devices for EVT have improved and EVT is becoming more widely used. However, the clinical outcome of EVT for Leriche syndrome remains unclear. A retrospective cohort study demonstrated that EVT for Leriche syndrome had a favorable outcome [4, 5].

There are a few case reports of simultaneous occurrence of DVT and Leriche syndrome [6]. From clinical course of this case, we speculated that Leriche syndrome had occurred firstly; after that, the left common iliac vein had been compressed by the left common iliac artery continuously and finally DVT had occurred. May-Thurner syndrome is an iliac vein compression syndrome in which compression of the left common iliac vein occurs by the overlying right common iliac artery [3]. In this case the left common iliac vein was compressed with the “left” common iliac artery. So this case was not exactly May-Thurner syndrome.

In this case, the tissue at the occlusion site in the terminal aorta seemed to be soft tissue from the CT findings, so we speculated that the occlusion was not so old. Furthermore, the patient had received abdominal surgery for intestinal obstruction about 20 years before. At that time the long hospital stay had been needed due to wound complications. And there was possibility of the adhesion of the abdominal organ. The adhesiolysis during reoperation was associated with an increase of sepsis incidence, intra-abdominal complications and wound infection, and longer hospital stay [7]. Therefore, we decided to treat Leriche syndrome with EVT. In this case, the terminal aorta was occluded with a massive thrombus. In such a case, distal embolization is one of the adverse complications. To prevent distal embolization, thrombus aspiration was done and dilatation after stenting was performed with small-size balloons. Fortunately, distal embolization did not occur in this case; however, it is important to take care of distal embolization. The clinical course of this case was excellent. So we concluded that EVT can be considered as a treatment option for Leriche syndrome.

## 4. Conclusion

We reported a case of Leriche syndrome accompanied with DVT treated with EVT for Leriche syndrome and the clinical course was excellent. EVT may be a treatment option for Leriche syndrome.
